# Genetic profiles of *Schistosoma haematobium* parasites from Malian transmission hotspot areas

**DOI:** 10.1186/s13071-023-05860-8

**Published:** 2023-08-04

**Authors:** Privat Agniwo, Jérôme Boissier, Bakary Sidibé, Laurent Dembélé, Assitan Diakité, Doumbo Safiatou Niaré, Ahristode Akplogan, Hassim Guindo, Manon Blin, Sarah Dametto, Moudachirou Ibikounlé, Thomas Spangenberg, Abdoulaye Dabo

**Affiliations:** 1 Department of Epidemiology of Infectious Diseases, Faculty of Pharmacy, Malaria Research and Training Center (MRTC), University of Sciences, Techniques and Technologies of Bamako, Environnement, Santé, Sociétés (USTTB/UCAD/UGB/CNRST/CNRS), BP 1805, IRL 3189 Bamako, Mali; 2 IHPE, University Montpellier, CNRS, Ifremer, University Perpignan Via Domitia, Perpignan, France; 3 Centre de Recherche pour la lutte contre les Maladies Infectieuses Tropicales (CReMIT/TIDRC), Université d’Abomey-Calavi, Abomey-Calavi, Bénin; 4 Global Health Institute of Merck, Ares Trading S.A., a subsidiary of Merck KGaA, Darmstadt, Route de Crassier 1, 1262 Eysins, Switzerland

**Keywords:** *Schistosoma haematobium*, *Schistosoma bovis*, *Schistosoma curassoni*, Hybridization, Cox1, ITS/18S, Mali

## Abstract

**Background:**

Although schistosomiasis is a public health issue in Mali, little is known about the parasite genetic profile. The purpose of this study was to analyze the genetic profile of the schistosomes of *Schistosoma haematobium* group in school-aged children in various sites in Mali.

**Methods:**

Urine samples were collected from 7 to 21 November 2021 and subjected to a filtration method for the presence *S. haematobium* eggs. The study took place in two schistosomiasis endemic villages (Fangouné Bamanan and Diakalèl), qualified as hotspots according to the World Health Organization (WHO) definition. Molecular genotyping on both Cox1 and ITS2/18S was used for eggs' taxonomic assignation.

**Results:**

A total of 970 miracidia were individually collected from 63 school-aged children and stored on Whatman FTA cards for molecular analysis. After genotyping 42.0% (353/840) and 58.0% (487/840) of miracidia revealed *Schistosoma bovis* and *S. haematobium* Cox1 profiles, respectively; 95.7 (885/925) and 4.3% (40/925) revealed *S. haematobium* and *S. haematobium/S. curassoni* profiles for ITS/18S genes, respectively. There was a significant difference in the Cox1 and ITS2/18S profile distribution according to the village (*P* < *0.0001*). Overall, 45.6% (360/789) were hybrids, of which 72.0% (322/447) were from Diakalèl. Three hybrids’ profiles (*Sb/Sc_ShxSc* with 2.3%; *Sb/Sc_ShxSh* with 40.5%; *Sh_ShxSc* with 2.8%) and one pure profile (*Sh_ShxSh* with 54.4%) were identified.

**Conclusion:**

Our findings show, for the first time to our knowledge, high prevalence of hybrid schistosomes in Mali. More studies are needed on population genetics of schistosomes at the human and animal interface to evaluate the parasite’s gene flow and its consequences on epidemiology of the disease as well as the transmission to humans.

**Graphical Abstract:**

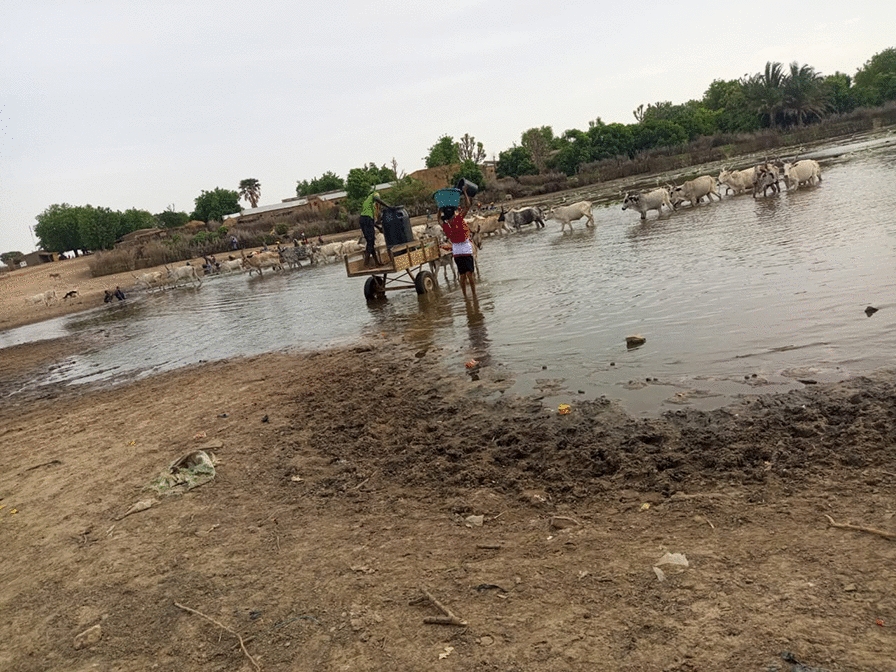

**Supplementary Information:**

The online version contains supplementary material available at 10.1186/s13071-023-05860-8.

## Introduction

Schistosomiasis is a parasitic disease of medical and veterinary importance that mainly affects tropical and subtropical areas. According to WHO [37], schistosomiasis affects almost 240 million people worldwide, with 85% of them living in Africa, and > 700 million people live in endemic areas. In Mali, the national prevalences of infection estimated in 2004–2006 were 38.3% and 6.7% for *Schistosoma haematobium* (*Sh*) and *S. mansoni* (*Sm*), respectively [10]. Whereas high *Sh* prevalence is widespread in all Malian regions, *Sm* infections are mainly restricted to small clusters in the center of the country (Macina and Niono districts in the Office du Niger irrigation area) and in Baguineda, 30 km from Bamako [8, 10, 33]. In addition to human vertebrate hosts, some *Schistosoma* species can also affect livestock. Overall, approximately 165 million parasitized animals worldwide are estimated to suffer from hemorrhagic enteritis, anemia and cachexia, and most often die [12]. In Africa, three *Schistosoma* species are involved in livestock infestation: *S. bovis* (*Sb*), *S. mattheei* (*Sma*) and *S. curassoni (Sc)* [23]. (*S.b*) is the most studied animal schistosome. *Sb* was first reported in Mali in 1988 in the Central Niger Delta where prevalence in animals is up to 80% [31]. Two years later, prevalences of 62.5% and 85.1% for *Sb* and *Sc* were reported in the slaughterhouses of Bamako and Mopti cities [26]. Beyond these cases reported in Mali, *Sb* occurs in the Mediterranean zone and throughout sub-Saharan Africa, especially in West Africa where it has been reported in almost all countries (Burkina Faso, Gambia, Ghana, Guinea, Bissau-Guinea, Mauritania, Niger, Nigeria, Senegal, Mali, Ivory Coast and Togo) [17, 21].

Hybridization is a biological phenomenon that corresponds to the meeting and crossing between two distinct genetic entities previously defined as different species [15, 20]. Hybridization can represent a real concern in terms of parasite transmission, epidemiology and morbidity. Hybridization in parasites can complicate the prevention, effective control and treatment of the disease [3] because hybrid forms sometimes exhibit higher virulence and/or resistance to treatments compared to their progenitor species [16, 30]. The study of hybridization has received renewed interest since the widespread use of molecular tools in parasite identification. Interestingly, eggs with typical (*Sb*) shape have been found in human feces [25], but to date no molecular tools are available to determine the hybrid status of these eggs. Today, natural hybridizations between schistosomes have already been identified, including between different species of animal-infecting schistosomes like *SbxSc* crosses in Senegal and Mali [26, 34], between human and animal-infecting schistosome species like *ShxSb* crosses in several West African countries [2, 14, 22, 27, 34] and between human-infecting schistosome *ShxSm* crosses in Senegal and Ivory Coast [13, 19].

In Mali, cases of hybridization have been reported in both animals and humans. In animals, cases of *ScxSb* hybrids were reported in cattle from slaughterhouses in Mopti and Bamako cities [26]. In humans, the only case of hybrid between *Sh* and *Sb* was reported in ten Belgian travelers who stayed on the Dogon Plateau, one of the most important endemic areas for *Sh* [29]. The hybrid status of the parasite was inferred by gene sequencing of two eggs recovered in the stool of patients. The nuclear ITS gene was assigned to *Sh* species while the Cox1 mitochondrial gene was assigned to *Sb* species [11].

The present study proposes the first epidemiological study to our knowledge on the possible presence of schistosome hybrids of the *S. haematobium* group in hyper-endemic areas of Mali.

## Methodology

### Site and period of study

**Fig. 1:**
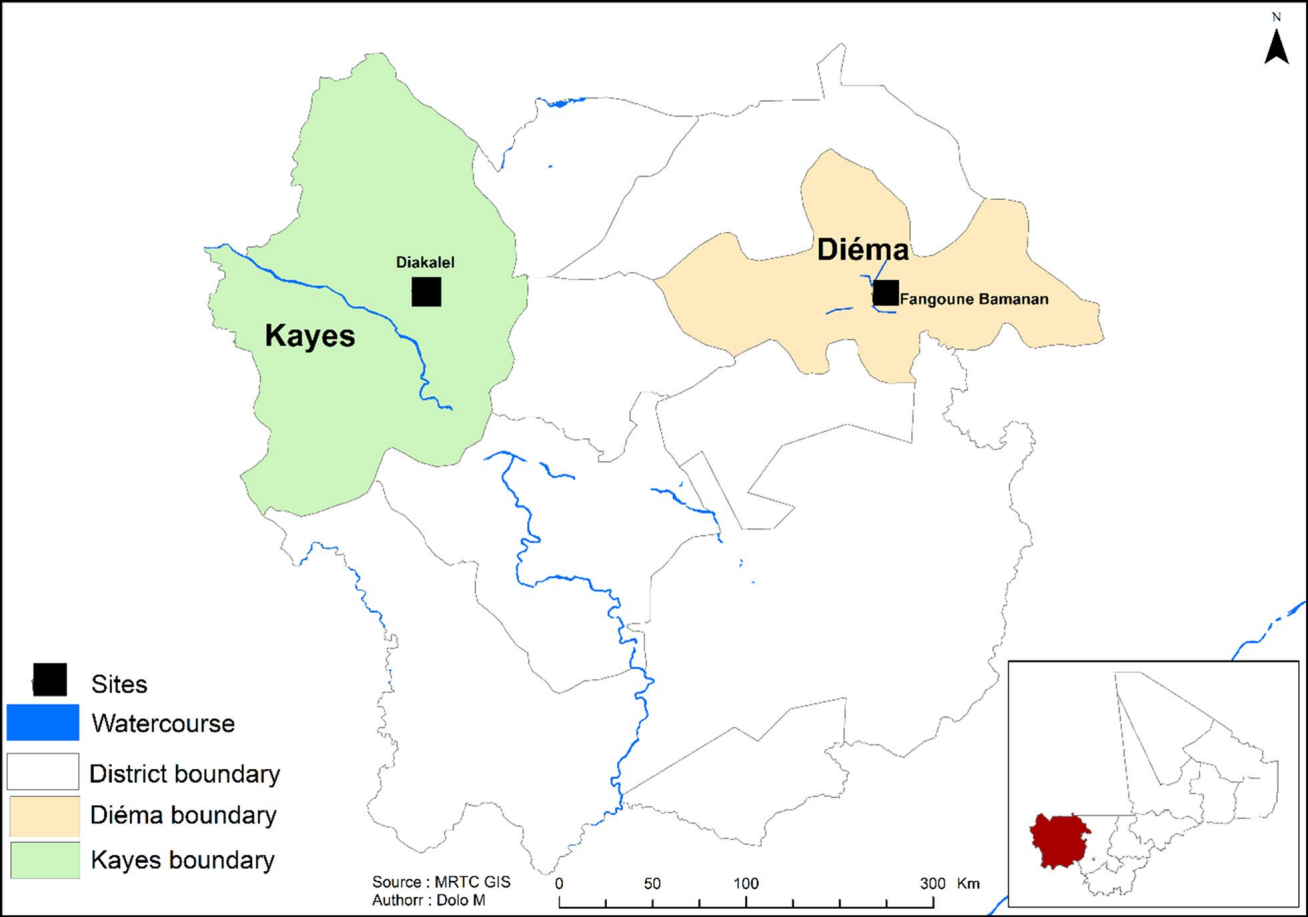
Kayes region (Mali, West Africa) and location of the two study sites

The study was conducted in November 2021 in two *Sh*-endemic villages (Fangouné Bamanan and Diakalèl) in the Kayes region of western Mali. These two villages are 300 km apart. They were chosen based on their proximity to water sources (ponds in the Diéma district, the Senegal River and its tributaries in the vicinity of the city of Kayes) (Fig. 1). The Kayes region is characterized by a northern Sudanese climate in the south and a Sahelian climate in the north with two main seasons: the rainy season (May–June to October), marked by average annual rainfall of up to 1000 mm in the south and 600 to 800 mm in the north, and the dry season, which extends from November to April–May [24]. Agriculture and livestock are the two main economic activities of the population [24]. The Sudano-Sahelian climate of the region is indeed favorable to the cultivation of dry cereals (millet, sorghum, maize) and groundnuts, and especially to extensive livestock farming where numerous herds of cattle, sheep and goats cohabit.

### Parasitological methods

Urine samples were collected from 393 children (251 in Diakalèl and 142 in Fangouné Bamanan) aged 6–14 years. Each child was assigned an identification number based on the first two letters of the village name. Urine samples were collected in 60-ml sterile jars between 9 a.m. and 2 p.m. After homogenization, 10 ml urine was taken with a syringe and filtered through a numbered Whatman filter paper (catalog no. 1001–025, 25 mm) previously placed in a filter holder. The filter was then stained with 3% ninhydrin, dried and rewetted with tap water before being read under a microscope with a 4× or 10× objective for *Sh* eggs. The eggs were counted and classified according to WHO [36] recommendation as light (1–49 eggs/10 ml) or heavy (≥ 50 eggs/10 ml) infection [36]. Urine samples from 63 heavily infected children were used for miracidia capture on FTA cards (GE Healthcare Life Sciences; Amersham, UK). Urine samples were filtered through a 40-µm filter (Fisherbrand^™^ Sterile Cell Strainers, USA, lot 100082019) and then rewashed in a watch glass using spring water. After 10 to 20 min contact with spring water, the miracidia started to hatch. Miracidia were individually collected using a P10 micropipette set to a volume of 3 µl and then captured on a FTA card. FTA cards were stored at room temperature in a ziplock bag protected from moisture until used for molecular analysis. A total of 30–35 miracidia were captured per child.

### Molecular analysis

Three-mm^2^ disks containing single miracidium were recovered from the FTA card using a Craft Punch and put in 1.5-ml Eppendorf tubes [2]. The DNA was then extracted using a previously published Chelex protocol [4]. A RD-PCR (rapid diagnostic multiplex PCR) was used to genotype the mitochondrial DNA Cox1 gene (mtDNA) [35], and ARMS (amplification-refractory mutation system-polymerase chain reaction) was used to simultaneously genotype the ITS2 and 18S nuclear genes [5]. RD-PCR produces two profiles (*Sh* and *Sb/Sc*) according to the band size (120 pb or 260 pb) in agarose gel. Note that the RD-PCR cannot differentiate between *Sb* and *Sc*. ARMS-PCR produces more complex profiles (4 to 6 bands) and allows discriminating *Sc*, *Sb*, *Sh* and all hybrid combinations. RD-PCR and ARMS-PCR genotyping methods were double checked by sequencing 136 parasite profiles. The resulting sequences were assembled and manually edited using Sequencher 4.5 (Gene Codes Corp.). Sequence polymorphisms were checked and confirmed by visualizing the chromatograms of the raw sequences and then aligned with the reference sequences using MEGA software. Then, the sequences were grouped by species and gene and aligned with previously published sequences with accession numbers. For the Cox1 gene (~ 1200 bp), the reference sequences for *Sh* were JQ397330.1 from Senegal and AY157209.1 from Mali, *Sb* (AJ519521.1 from Senegal and MT159594.1 from Benin) and *Sc*) (MT579424.1 and MT579422.1 from Senegal. We compared our sequences with those of *Sh*, *Sb* and *Sc*, already published in Genbank with the following respective accession numbers: GU257398.1, MT580950.1 and MT580946.1 for ITS (~ 630) and Z11976.1, AY157238.1 and AY157236.1 for 18S (~ 330). Species-specific SNPs allowed us to differentiate them [5]. Combining the species assignment of the mitochondrial and nuclear genes gives the following possible genotypes: *Sb_SbxSb; Sh_ShxSh; Sc_ScxSc* (considered pure) and *Sb_SbxSh; Sb_SbxSc; Sh_ShxSb; Sh_ShxSc; Sc_ScxSh* and *Sc_ScxSb* (considered hybrid).

### Statistical analysis

Gene proportions were tested using the Chi-square or Fisher’s exact tests depending on the data. Probability values (*P*) < 0.05 were considered statistically significant.

### Ethical approval

The project was reviewed and approved by the Institutional Review Board (IRB) of the Faculty of Medicine, Pharmacy and Dentistry of the University of Bamako (no. 2018/71/CE/FMPOS). Community consent was obtained before the study began. Only children whose parents or guardians and who themselves accepted to participate in the study were registered and requested to provide urine samples. Individuals who tested positive for schistosomiasis infection were treated with praziquantel (40 mg/kg of body weight) according to the Schistosomiasis National Control Program (PNLSH) guideline.

## Results

A total of 393 urine samples were examined for *Sh* egg diagnostic (Table 1). The overall prevalence was 69.2% (272/393). The prevalence and intensity of infection were significantly higher in Diakalèl compared to Fangouné Bamanan (*P* < *0.0001).* Urine from 63 heavily positive children (48 in Diakalèl and 15 in Fangouné Bamanan) was used to collect and store miracidia on FTA cards.

**Table 1:** Prevalence and intensity of *Schistosoma haematobium* in the two study sites

**Villages**	**Number of children examined**	**Positive**	**Prevalence (%)**	**P-value**	**Negative (%)**	**Light infection (%)**	**Heavy infection (%)**	**P-value**
Diakalèl	251	196	78.1	< 0.0001	55 (21.9)	148 (59.0)	48 (19.1)	< 0.0001
Fangouné Bamanan	142	76	53.5		66 (46.5)	61 (43.0)	15 (10.5)	
Total	393	272	69.2		121 (30.8)	209 (53.2)	63 (16.0)	

A total of 970 samples were genotyped, from which only 840 gave consistent results using the RD-PCR (Table 2 and Additional file 1: Table S1). From these 840 samples, 353 (42.0%) showed a *Sb/Sc* band size, and 487 (58.0%) showed a *Sh* band size. Fifty-five samples from 353 (about 15%) randomly chosen (47 from Diakalèl and 8 from Fangouné Bamanan) with *Sb/Sc* profile were sequenced, and all showed a *Sb* profile. Forty samples on 487 (8%) randomly chosen (17 from Diakalèl and 23 from Fangouné Bamanan) with *Sh* profile were sequenced, and all showed a *Sh* profile. The relative repartition of each haplotype was different between Diakalèl and Fangouné Bamanan with three times more *Sh* haplotype in Fangouné Bamanan compared to Diakalel. Of 970 genotyped samples, 925 gave consistent results using the ARMS-PCR, of which 885 (95.7%) showed a *ShxSh* profile and 40 (4.3%) a *ShxSc* profile (Table 2). Twenty-six samples of 885 (3%) randomly chosen (7 from Diakalèl and 19 from Fangouné Bamanan) with *ShxSh* profile were sequenced, and all showed a *ShxSh* profile. Fifteen samples of 40 (37.5%) randomly chosen (10 from Diakalèl and 5 from Fangouné Bamanan) with *ShxSc* profile were sequenced, and all showed a *ShxSc* profile.

**Table 2:** Number and percentage (in parentheses) of genes identified by rapid diagnostic PCR (RD_PCR) for Cox 1, ARMS_PCR for IT2/18S and parasite genetic profiles in the two *Schistosoma haematobium* parasites collected in two villages in Mali

			**Haplotype Cox1**				**Genotype ITS/18S**								
**District**	**Village**	**Total**	**Sb/Sc**	**Sh**	< **0. 0001**	**Total**	**ShxSh**	**ShxSc**	= **0.14**	**Total**	**Sb/Sc_ShxSc**	**Sb/Sc_ShxSh**	**Sh_ShxSc**	**Sh_ShxSh**	**Total hybrides**
Kayes	*Diakalèl*	475 (56.5)	333 (70.0)	142 (30.0)		526 (56.9)	508 (96.6)	18 (3.4)		447	16 (3.6)	304 (68.0)	2 (0.4)	125 (28.0)	322 (72.0)
Diéma	*Fangouné Bamanan*	365 (43.5)	20 (5.5)	345 (94.5)		399 (43.1)	377 (94.5)	22 (5.5)		342	2 (0.6)	16 (4.7)	20 (5.8)	304 (88.9)	38 (11.1)
	*Total*	840	353 (42.0)	487 (58.0)		925	885 (95.7)	40 (4.3)		789	18 (2.3)	320 (40.5)	22 (2.8)	429 (54.4)	360 (45.6)

There was a significant difference in the Cox1 profile distribution between the two villages (*P* < *0.0001*). The frequency of *Sh* ITS2/18S alleles was > 90% in both villages. The distribution of *ShxSh* and *ShxSc* genotypes was comparable between the two villages (*P* = *0.14*) (Table 2). Four genetic profiles were identified after genotyping miracidia using Cox1 and ITS/18S: *Sb/Sc* cox1 × *Sh* ITS 2/*Sc*_18S (*Sb/Sc_ShxSc); Sb/Sc*_cox1 × *Sh*_ITS 2/* Sh* _18S (*Sb/Sc_ShxSh); Sh*_cox1 × *Sh*_ITS2/*Sc* _18S (*Sh_ShxSc)*; *Sh*_cox1 × *Sh*_ITS 2/*Sh* _18S (*Sh_ShxSh)*. Three hybrid profiles [*Sb/Sc_ShxSc* (2.3%); *Sb/Sc_ShxSh* (40.5%); *Sh_ShxSc* (2.8%)] and one pure genetic profile [*Sh_ShxSh* (54.4%)] have been described. Among the three hybrid profiles, the *Sb/Sc_ShxSh* profile was more widely represented in Diakalèl compared to Fangouné Bamanan (68.0% vs. 4.7%). In contrast, the non-hybrid *Sh_ShxSh* genetic profile was more common in Fangouné Bamanan (88.9%) (Table 2: Additional file 1: Table S1). Overall, 45.6% (360/789) of hybrids were recorded. The highest prevalence of hybrids was observed in Diakalèl with 72.0%.

## Discussion

We carried out parasitological and molecular study focused on collecting urine from children from a schistosomiasis-endemic area subjected to several years of mass drug treatment (MDT) with PZQ. With an average *S*. *haematobium* prevalence of 69.2%, the rate is higher than that recently reported in six villages in the Kayes region (50.1%) [1]. The prevalence and intensity were significantly higher in Diakalèl compared to Fangouné Bamanan (*P* < *0.0001*). This difference between the villages could be explained by the type of watercourse, the Senegal River and its tributaries in Diakalel being more frequented than ponds in Fangouné Bamanan.

The molecular analysis allowed determining the genetic profiles of both a mitochondrial (Cox1) and two nuclear (ITS2 and 18S) genes. Of the 840 miracidia genotyped, which gave consistent results using the RD-PCR in both villages, (42.0%) and (58.0%) had *Sb/Sc* and *Sh* Cox1 profiles, respectively. RD-PCR can discriminate *Sh* and *Sm* but cannot differentiate *Sb* from *Sc*. Although 15% of the randomly selected sequences were identified as *Sb* regardless of nuclear profile, we suppose that in the overall sampling, the vast majority of Cox1 came from *Sb* and not *Sc*. The percentage of *Sh* (58.0%) we found was higher than those observed in Nigeria (11%) [22] and Ivory Coast (49.3%) [2] but lower than those described in Senegal (79.0%) [7] and Cameroon (95.8%) [32]. For ITS2/18S genes, two profiles were identified with 95.7% for *ShxSh* and 4.3% for *ShxSc*. This percentage of *ShxSh* is higher than observed in Nigeria (40.7%) [22] and Ivory Coast (87.9%) [2] but lower than found in Senegal (97.9%). [7] and Cameroon (92.5%) [32]. All these studies showed that *Sh* nuclear alleles are dominant in parasites collected from humans.

Analysis of Cox1 and ITS2/18S genetic profiles was performed on 789 miracidia, of which 360 (45.6%) yielded three types of hybrids: *Sb/Sc_ShxSc*, *Sb/Sc_ShxSh* and *Sh_ShxSc*. This percentage of hybrids is lower than those observed in Ivory Coast (62.7%) [2] and Nigeria (89%) [22] but higher than that observed in Cameroon (11.3%) [32]. The hybrids with *Sb/Sc_ShxSc* profiles indicate gene flow among the three different schistosome species. The observation of the three-species hybrid (Sb_ShxSc) in our study suggests the possibility of interactions and pairing between these species as previously reported by [18] in Niger. Such results confirm the potential for repeated interactions and cross-pairing among these three species and support the hypothesis that they are not first-generation hybrids but rather relatively recent parental and/or hybrid backcrossing [18]. The hybrid genotype profile observed in our study could be explained by the use of the same water points by human populations and animals, especially in the Sahel where water scarcity is a recurring phenomenon. Beyond the hybridization between *Sb* and *Sh* previously shown by several authors [2, 14, 22, 27, 29, 32], we also observed *Sh_ShxSc* hybrids in children, suggesting clearly that *Sh* can hybridize naturally with *Sc*, as has also been shown in Senegal [34]. The absence of samples from animals is the main limitation of this study. Genotyping of miracidia from livestock or rodents could provide information on the zoonotic transmission as was the case in Benin with cows [28] and rodents [27] and only in rodents in Senegal [9]. Our research can be considered a pilot study that needs to be replicated across the country to confirm or not the frequency of the three hybrid *Sb_ShxSc* species.

## Conclusion

Our results showed, for the first time to ouir knowledge, high prevalence of hybrid schistosomes in human infections in the Kayes region of Mali. The presence of hybrids underscores the importance of the livestock reservoir of schistosomes, hampering schistosomiasis control and elimination. More studies are needed on population genetics of schistosomes at the human and animal interface to evaluate the parasite’s gene flow and its impact on epidemiology of the disease, transmission to humans and disease control.

### Supplementary Information

**Additional file 1. Table S1**: Genetic data of parasite per child and sentinel site.

## Data Availability

All data and materials are available in the article.

## Abbreviations

*S*: *Schistosoma*
*h*: *haematobium*
*c*: *curassoni*
*b*: *bovis*
WHO: World Health Organization
MDA: Mass Drug Administration
FTA: Find the agent
ITS: Internal transcribed spacer
ARMS: Amplification refractory mutation system
CAT: Catalogue
DNA: Deoxyribonucleic acid
mt: Mitochondrial
IR: Interne reverse
IF: Interne forward
OR: Out reverse
OF: Out forward
rDNA: Recombinant deoxyribonucleic
SRB: Senegal River basin
