# Publisher Correction: Genetic profiles of *Schistosoma haematobium* parasites from Malian transmission hotspot areas

**DOI:** 10.1186/s13071-023-06061-z

**Published:** 2023-12-15

**Authors:** Privat Agniwo, Jérôme Boissier, Bakary Sidibé, Laurent Dembélé, Assitan Diakité, Doumbo Safiatou Niaré, Ahristode Akplogan, Hassim Guindo, Manon Blin, Sarah Dametto, Moudachirou Ibikounlé, Thomas Spangenberg, Abdoulaye Dabo

**Affiliations:** 1 Department of Epidemiology of Infectious Diseases, Faculty of Pharmacy, Malaria Research and Training Center (MRTC), University of Sciences, Techniques and Technologies of Bamako, IRL 3189 Environnement, Santé, Sociétés (USTTB/UCAD/UGB/CNRST/CNRS), BP 1805, Bamako, Mali; 2 IHPE, University Montpellier, CNRS, Ifremer, University Perpignan Via Domitia, Perpignan, France; 3 Centre de Recherche Pour La Lutte Contre Les Maladies Infectieuses Tropicales (CReMIT/TIDRC), Université d’Abomey-Calavi, Abomey‑Calavi, Bénin; 4 Global Health Institute of Merck, Ares Trading S.A., a Subsidiary of Merck KGaA, Darmstadt, Route de Crassier 1, 1262 Eysins, Switzerland

**Publisher Correction: Parasites & Vectors (2023) 16:263** **https://doi.org/10.1186/s13071-023-05860-8**

Following publication of the article [1], it came to the journal's attention that, with the exception of the sixth author, the respective given and family names of the authors were the wrong way around. The author list has since been corrected in the published article. The publisher thanks you for reading this erratum and apologizes for any inconvenience caused by this processing error.

## References

[1] Agniwo P, Boissier J, Sidibé B, Dembélé L, Diakité A, Niaré DS, Akplogan A, Guindo H, Blin M, Dametto S, Ibikounlé M, Spangenberg T, Dabo A (2023). Genetic profiles of* Schistosoma haematobium* parasites from Malian transmission hotspot areas. Parasit Vectors.

